# Cytological biomarkers and risk of malignancy in indeterminate thyroid nodules: a systematic review and meta-analysis

**DOI:** 10.1530/EO-25-0097

**Published:** 2026-09-04

**Authors:** Trille Moberg Fagerhaug, Mathilde Hvidtfelt Kjeldsen, Mathias Maagaard, Preben Homøe, Gitte Bjørn Hvilsom

**Affiliations:** ^1^ Department of Otorhinolaryngology, Head and Neck Surgery, Zealand University Hospital, Køge, Denmark; ^2^ Department of Anaesthesiology and Intensive Care, Zealand University Hospital, Køge, Denmark; ^3^ Department of Clinical Medicine, University of Copenhagen, Copenhagen, Denmark

**Keywords:** thyroid, FNAC, Galectin-3, HBME-1, BRAF

## Abstract

**Purpose:**

Thyroid nodules with indeterminate cytology are often referred for diagnostic surgery. This systematic review evaluated diagnostic markers in fine-needle aspiration cytology samples of indeterminate nodules to improve differentiation between benign and malignant nodules.

**Methods:**

We searched MEDLINE, Embase, and the Cochrane Library from 1995 to 2025. The literature was screened, and data were extracted from the included studies. The quality of studies was assessed with QUADAS-2. We performed split component meta-analysis of diagnostic accuracy using R-studios and the SCS meta-function (split component synthesis).

**Results:**

Forty-one relevant studies were identified, of which 23 eligible studies were included in the meta-analysis. Data on Galectin-3, HBME-1, and BRAF mutation from 1,564 patients were evaluated. Galectin-3 and HBME-1 demonstrated promising diagnostic accuracy, with diagnostic odds ratios (DORs) of 20.3 (95% CI: 9.7–42.5) and 15.0 (95% CI: 7.3–31.0), sensitivities of 0.74 (95% CI: 0.64–0.82) and 0.77 (95% CI: 0.67–0.84), and specificities of 0.88 (95% CI: 0.80–0.93) and 0.82 (95% CI: 0.73–0.89), respectively. Combined positivity yielded the highest DOR of 45.8 (95% CI: 17.7–118.6), sensitivity of 0.90 (95% CI: 0.81–0.95), and specificity of 0.84 (95% CI: 0.73–0.91). BRAF mutation had the lowest DOR of 6.6 (95% CI: 2.1–20.4) and sensitivity of 0.17 (95% CI: 0.09–0.29) but the highest specificity of 0.97 (95% CI: 0.93–0.99).

**Conclusion:**

Our meta-analysis indicates that Galectin-3 and HBME-1, particularly when combined, may improve differentiation between benign and malignant nodules. BRAF mutation is highly specific but lacks sensitivity. These biomarkers show potential, but none warrant stand-alone clinical use, emphasizing the need for large-scale validation and potentially a multi-marker approach to improve diagnostic accuracy.

## Introduction

Thyroid nodules are a common clinical finding, detectable in approximately 19–35% of the adult population through ultrasound examination, approximately three times more frequent among women (1, 2). The majority of thyroid nodules are benign, asymptomatic, and frequently incidentally identified during a radiological procedure performed for unrelated reasons. The risk of malignancy in a thyroid nodule is approximately 7–15% (3). Differentiating between benign and malignant nodules represents an important clinical challenge, with ultrasound characteristics and fine-needle aspiration cytology (FNAC) as the cornerstones in evaluating the nodules. Ultrasound-guided FNAC of the thyroid gland is a well-established diagnostic technique for the preoperative evaluation of thyroid nodules, allowing a significant reduction in the number of diagnostic thyroidectomies by distinguishing benign and malignant nodules (4). Correct cytopathological interpretation allows for classification into diagnostic categories according to The Bethesda System for Reporting Thyroid Cytopathology (TBSRTC), an internationally recognized system introduced in 2010 for stratifying risk of malignancy in thyroid nodules (5).

TBSRTC classifies thyroid nodules into six categories, each associated with an increasing risk of malignancy, thereby ensuring appropriate clinical management, including necessity of surgical intervention. However, up to 30% of FNAC specimens yield indeterminate cytological diagnosis, posing a significant diagnostic challenge (6). The specificity of FNAC is especially limited in the evaluation of TBSRTC category III and IV nodules, since cytology alone cannot differentiate between follicular thyroid adenomas (FTAs) and malignant follicular thyroid carcinomas (FTCs) (6). Therefore, patients with thyroid nodules diagnosed with TBSRTC category III, IV, or other classifications of indeterminate cytology, are typically selected for diagnostic hemi- or total thyroidectomy. Nevertheless, only 6–18% of TBSRTC category III nodules and 10–40% of TBSRTC category IV nodules are ultimately found to be malignant. These malignancy rates, although relatively low, pose sufficient diagnostic uncertainty to warrant surgical intervention for definitive histological confirmation (6).

To limit the number of unnecessary surgeries of benign nodules while ensuring appropriate oncologic management and sufficient surgical treatment for patients with thyroid carcinoma, a more precise cytological diagnostic approach is required. Due to the diagnostic limitations of FNAC, no reliable method currently exists for the preoperative identification of malignancy in follicular lesions of the thyroid gland.

Efforts have been made to assess the potential of biomarkers in differentiating benign from malignant indeterminate thyroid nodules. Enhancing preoperative diagnostic accuracy would benefit both patients and healthcare systems, as it would reduce the frequency of unnecessary surgeries of benign nodules and decrease the risk of surgical complications.

The aim of the present systematic review and meta-analysis is to evaluate and summarize the existing literature on diagnostic accuracy of molecular biomarkers in preoperative FNAC of indeterminate thyroid nodules prior to surgery in terms of distinguishing between benign and malignant thyroid nodules.

## Methods

### Protocol and registration

A protocol was drafted according to Preferred Reporting Items for Systematic reviews and Meta-Analysis Protocols (PRISMA-P) (7). The final protocol was registered in PROSPERO (registration number: CRD42023423138).

### Information sources and search

We performed an electronic database search of MEDLINE, Embase, and the Cochrane Library to identify studies on presurgical FNAC samples of the thyroid gland with indeterminate cytology. The searches were made using the Medical Subject Headings (MeSH) terms ‘Thyroid Gland’, ‘Immunohistochemistry’, ‘Biomarkers’, and variants of these. No language restrictions were applied during the literature search. The electronic databases were last searched on January 16, 2025.

The search strategy was discussed with team members and was finally peer-reviewed by an information specialist. The final search strategy for each database can be found in supplementary material. Search results were exported to Zotero (https://www.zotero.org/), a citation manager, and then uploaded to Covidence (https://www.covidence.org/), an online tool for screening and data extraction.

Additional potentially relevant studies were identified in reference lists of eligible studies and relevant reviews and meta-analysis.

The search results were initially screened by title and abstract by three authors (TM, MK, and GBH), which resulted in exclusion of articles that were not original studies or in the field of interest. Next, full-text screening of the remaining studies was carried out by two authors (TM and MK) independently and in duplicate. Any discrepancies between the reviewers were solved through discussion; if consensus could not be reached, a third reviewer was consulted (GBH).

The results of this systematic review and meta-analysis were reported according to the Cochrane Handbook for Systematic Reviews of Diagnostic Test Accuracy (8).

### Study selection and eligibility criteria

The following criteria were used for the inclusion and exclusion of studies in this review:

#### Inclusion criteria:

- Original studies reporting on FNAC specimens of patients with the cytological diagnosis corresponding to follicular lesions of undetermined significance or follicular neoplasm and known postoperative histopathological diagnosis.
- Published between 1995 and 2025.

#### Exclusion criteria:

- Case reports, case series, reviews, editorials, and letters to editor.
- Animal studies.
- Pediatric studies.
- Studies that otherwise met the inclusion criteria but lacked accessible data for direct extraction or by correspondence with author.

To ensure sufficient statistical power and reduce sparse-data bias, a minimum of four independent studies per biomarker was predefined as a pragmatic threshold for quantitative synthesis. Biomarkers reported in fewer studies were synthesized narratively and are reported in supplementary material.

### Data extraction

Data on expression of biomarkers were extracted from nodules with preoperative indeterminate FNAC diagnosis. The marker expressions and test performances were compared with the postoperative histopathological diagnosis of the thyroid nodules.

Data were extracted by two reviewers (TM and GBH) independently and in duplicate, which included study information (study design, period of study, and sample size), details on index test, conventional FNAC diagnosis, and results of postsurgical histopathology diagnosis and outcome (true positive, true negative, false positive, and false negative). Discordance between the two reviewers was resolved by discussion or by consulting with a third reviewer (PH).

### Quality assessment

The Quality Assessment of Diagnostic Accuracy Studies-2 (QUADAS-2) tool (9) was used to evaluate the quality of included studies by two reviewers (TM and GBH) independently and in pair. The QUADAS-2 tool consists of four key domains (patient selection, index test conduct and interpretation, reference standard, and patient flow and timing). Each domain was rated for each study independently, according to risk of bias and applicability to the review question. Discordance between the two reviewers was resolved by discussion or by consulting with a third reviewer (PH).

### Synthesis of results and statistical analysis

The meta-analysis was performed using R-studio (https://posit.co/products/open-source/rstudio) and Review Manager 5.4 (https://revman.cochrane.org/).

Data on true positives, false positives, true negatives, and false negatives were extracted from the studies evaluating the expression of Galectin-3, HBME-1, BRAF, and Galectin-3/HBME-1 co-expression. These were used to estimate the pooled diagnostic odds ratio (DOR) with 95% confidence interval (CI) using the inverse-variance heterogeneity meta-analysis model.

Heterogeneity was quantified using the *I*^2^ statistic calculated on the log-transformed diagnostic odds ratio (log DOR). *I*^2^ has traditionally been used as a measure of heterogeneity. However, *I*^2^ has important limitations in the context of meta-analysis of diagnostic test accuracy. First, *I*^2^ is not a direct measure of between-study heterogeneity, but rather a measure of the proportion of between-study variation not explained by chance. The estimation of *I*^2^ does not take into account the source of between-study variation. Second, *I*^2^ is influenced by the number of studies and sample size. Increasing sample size tends to inflate *I*^2^ without actual increases in between-study variability, and *I*^2^ might also be low due to a lack of power to detect actual differences. Threshold effects were assessed using Spearman rank correlation between logit-transformed sensitivity and false-positive rate.

We then applied the split component synthesis method for estimating the pooled area under the curve, pooled sensitivity, pooled specificity, and pooled positive and negative likelihood ratios. The centered DOR (study specific DOR ‘minus’ the pooled DOR) was used to determine the pooled sensitivity and specificity using ordinary least squares regression, where the intercepts of the regression models correspond to the pooled sensitivity and pooled specificity. The results are presented as a descriptive forest plot with raw data, sensitivity, and specificity for each study and as a summary receiver operating characteristic (SROC) plot.

The conventional bivariate model, which in the absence of covariates is equivalent to the hierarchical summary receiver operating characteristic (HSROC) model, has been the meta-analytic model of choice for modeling diagnostic accuracy across studies. The bivariate model jointly models sensitivity and specificity through hierarchical random-effects structures. The input to the bivariate model is the sensitivity and specificity of each study, which are prone to between-study heterogeneity due to various reasons, including design errors and variability in test thresholds. The bivariate model is at risk of performing poorly in the presence of increasing between-study heterogeneity, decreasing number of studies, and variations in threshold variability. The SCS method first pools the diagnostic odds ratio (DOR) and subsequently derives pooled sensitivity and specificity through regression on the centered DOR. The DOR is a measure of test discrimination, which is relatively immune to bias from threshold variability across a wide range of commonly used thresholds. Therefore, basing meta-analysis on the DOR and subsequently estimating the pooled sensitivity and specificity of a test has been suggested to be more robust than basing the meta-analysis on the study-specific sensitivity and specificity. A simulation study from 2021 suggested that the method of split component synthesis (SCS) was less biased than the bivariate model estimator, previously used for synthesizing test accuracy data (10), which is why we chose to use this test strategy.

## Results

### Literature search

**Figure 1:**
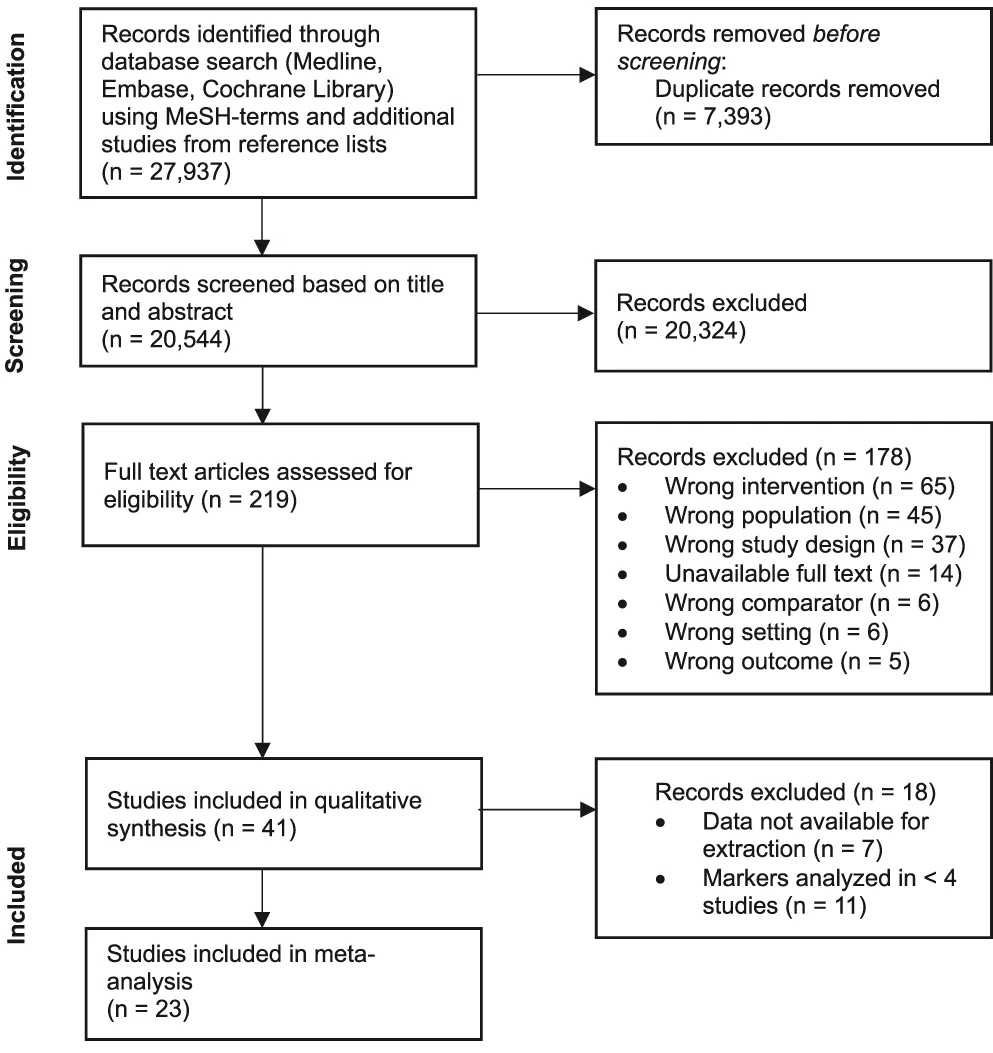
PRISMA flow chart of selection process for studies included in meta-analysis.

Out of 27,937, we ended up including 41 studies that met the criteria for inclusion (see Fig. 1). Data were extracted from 23 studies reporting on three different markers, all of which were included in the meta-analysis (Table 1).

**Table 1:** Included studies with molecular biomarkers, preoperative fine-needle aspiration cytology, and final histopathological benign or malignant diagnosis of indeterminate thyroid nodules.

Study	Marker	Preoperative diagnosis	Postoperative diagnosis
Abram *et al.* (11)	Galectin-3, HBME-1	64 AUS/FLUS	47 B, 17 M
Addati *et al.* (12)	HBME-1	63 AUS/FLUS, 5 FN/SFN	56 B, 12 M
Anand *et al.* (13)	BRAF	27 FN/SFN	17 B, 10 M
Barros-Filho *et al.* (14)	BRAF	8 AUS/FLUS and FN/SFN	4 B, 4 M
Bartolazzi *et al.* (15)	Galectin-3	432 indeterminate – THY3	302 B, 130 M
Borrelli *et al.* (16)	BRAF	30 AUS/FLUS and FN/SFN	16 B, 14 M
Cha *et al.* (17)	HBME-1	26 AUS/FLUS	6 B, 20 M
Collet *et al.* (18)	Galectin-3	34 indeterminate*	22 B, 12 M
Decaussin-Petrucci *et al.* (19)	BRAF	61 AUS/FLUS, 124 FN/SFN	152 B, 33 M
Dell’Aquila *et al.* (20)	BRAF	75 AUS/FLUS and FN/SFN	61 B, 14 M
Dixit *et al.* (21)	Galectin-3	8 AUS/FLUS, 10 FN/SFN	7 B, 11 M
Elsharkawy *et al.* (22)	Galectin-3	25 FN	13 B, 12 M
Fadda *et al.* (23)	Galectin-3, HBME-1	50 AUS/FLUS and FN/SFN	39 B, 11 M
Gill *et al.* (24)	BRAF	60 AUS/FLUS and FN/SFN	37 B, 23 M
Leslie *et al.* (25)	HBME-1, BRAF	4 AUS/FLUS, 3 FN/SFN	1 B, 7 M
Mills *et al.* (26)	Galectin-3	4 indeterminate – THY3	1 B, 3 M
Pennelli *et al.* (27)	Galectin-3	100 FN	85 B, 15 M
Rossi *et al.* (28)	Galectin-3, HBME-1	37 follicular lesions*	20B, 17 M
Rossi *et al.* (29)	Galectin-3. HBME-1	164 AUS/FLUS and FN/SFN	133 B, 31 M
Rossi *et al.* (30)	Galectin-3, HBME-1, BRAF	37 AUS/FLUS and FN/SFN	20 B, 17 M
Sapio *et al.* (31)	Galectin-3, BRAF	20 indeterminate*	18 B, 2 M
Torregrossa *et al.* (32)	Galectin-3, HBME-1	43 indeterminate*	28 B, 15 M
Zhang *et al.* (33)	Galectin-3, HBME-1	50 AUS/FLUS	28 B, 22 M

^*^ Not further specified.

AUS, atypia of undetermined significance; B, benign; FLUS, follicular lesion of undetermined significance; FN, follicular neoplasm; M, malignant; SFN, suspicious of follicular neoplasm.

### Characteristics and quality assessment of selected studies

For the meta-analysis, 23 original articles were included, comprising 12 prospective and 11 retrospective studies. All types of indeterminate thyroid nodules were considered, including those classified as Bethesda categories III and IV, THY3 in the British Thy classification system (34), or otherwise labeled as indeterminate. In total, data from 1,564 patients with a preoperative diagnosis of an indeterminate thyroid nodule and postoperative histopathology diagnoses were analyzed.

The majority of studies provided detailed methodological information on FNAC technique, needle gauge, immunocytochemistry staining protocols, and scoring criteria. The markers included in the meta-analysis were Galectin-3, HBME-1, and BRAF, evaluated in 14, 10, and 9 studies, respectively. Furthermore, five studies evaluating co-expression of Galectin-3 and HBME-1 were included. These studies had comparable cutoffs for positive and negative results. Data from studies not meeting criteria for inclusion in meta-analysis can be found in Supplementary Table S1 (see section on Supplementary materials given at the end of the article).

**Figure 2:**
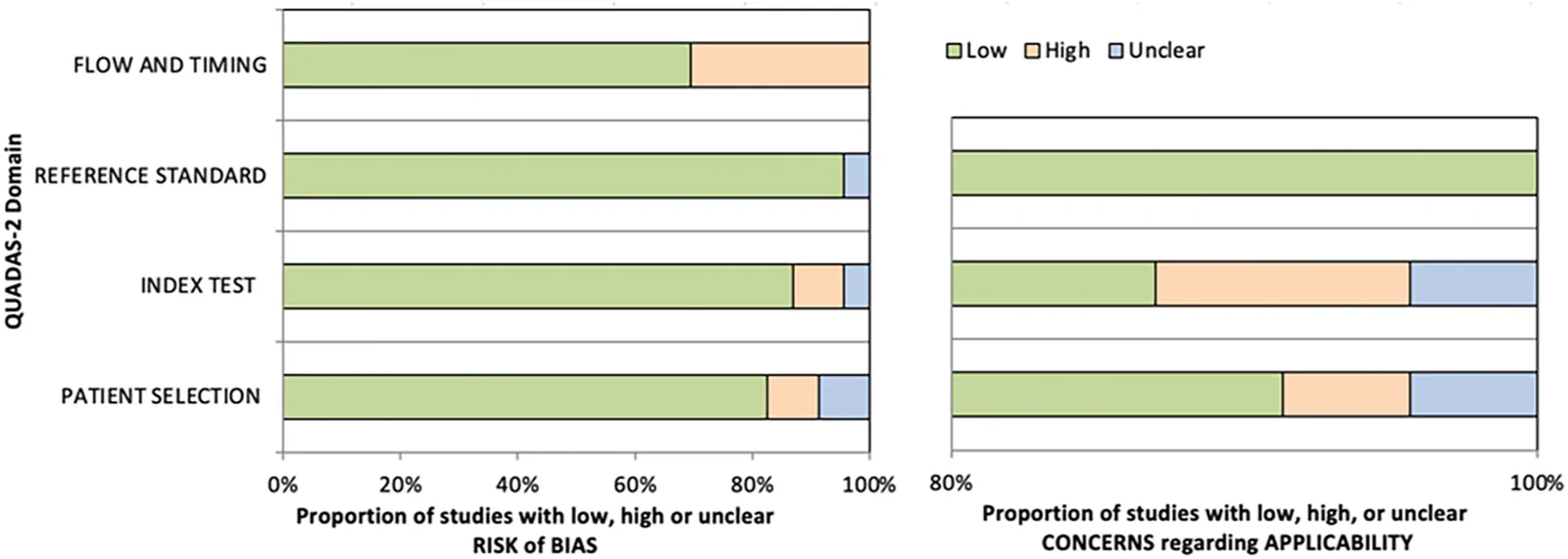
Quality analysis of 23 studies included in meta-analysis according to QUADAS-2 criteria.

The quality of the included studies was assessed using QUADAS-2 criteria, evaluating both risk of bias and study applicability. A summary of the quality assessments is presented in Fig. 2. While the overall quality of the studies was acceptable, flow and timing, patient selection, and index test were identified as the primary sources of potential bias.

### Threshold effect assessment

No statistically significant threshold effects were detected for any of the evaluated markers. The correlation coefficients were *ρ* = 0.53 (*P* = 0.052) for Galectin-3, *ρ* = 0.49 (*P* = 0.15) for HBME-1, *ρ* = 0.03 (*P* = 0.93) for BRAF, and *ρ* = 0.8 (*P* = 0.13) for the Galectin-3/HBME-1 combined panel. Detailed information on immunocytochemistry scoring cutoffs and BRAF detection methods is provided in Supplementary Table S2.

### Marker diagnostic performance

The diagnostic accuracy of the markers (Galectin-3, HBME-1, and BRAF) in distinguishing indeterminate thyroid nodules as benign or malignant was evaluated.

**Figure 3:**
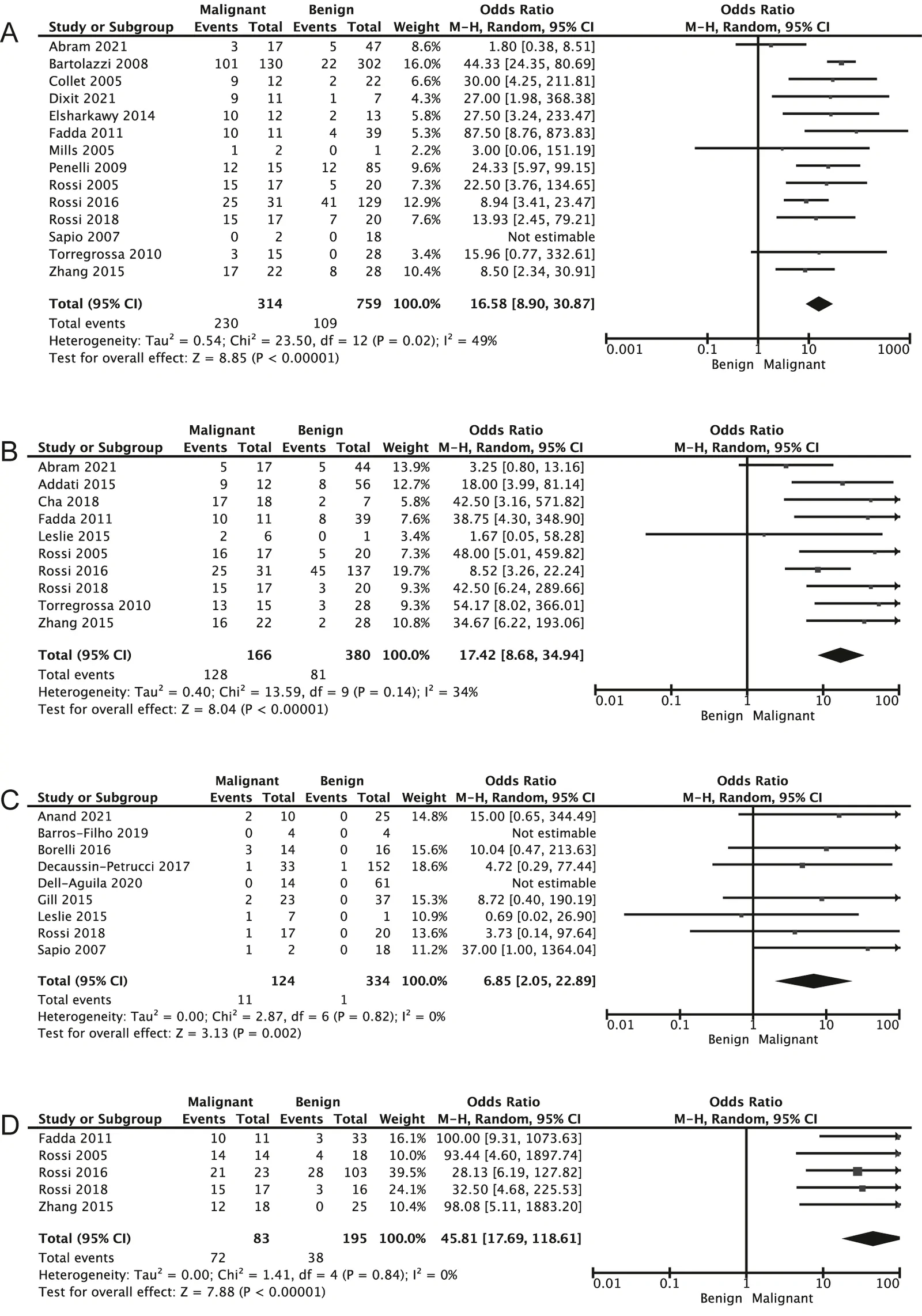
Descriptive forest plot showing DOR of markers differentiating benign from malignant nodules with indeterminate FNAC samples. (A) Galectin-3, (B) HBME-1, (C) BRAF, and (D) Galectin-3/HBME-1 co-expression.

**Figure 4:**
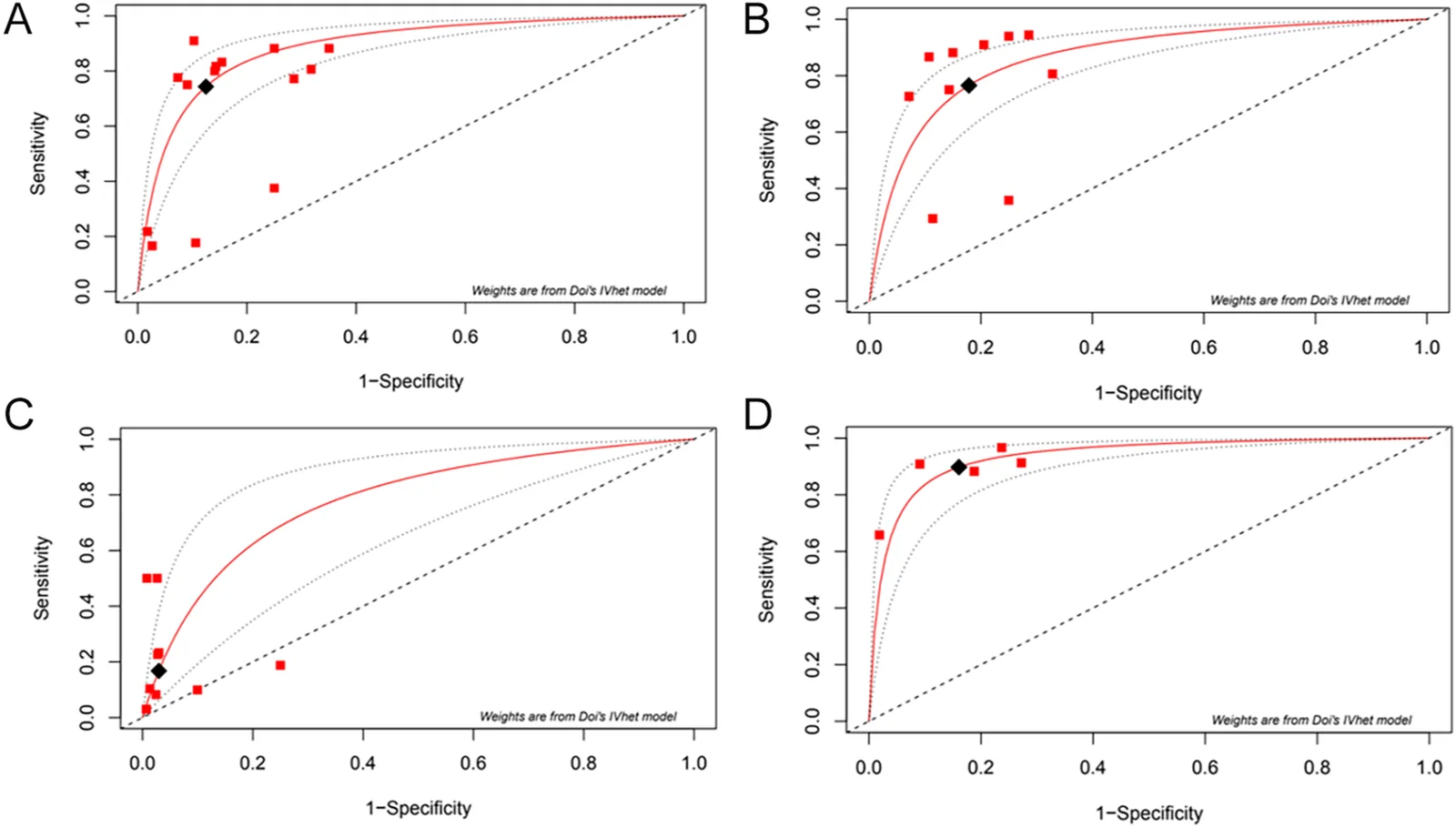
Summarized receiver operating characteristics (SROC) plot of the meta-analysis of (A) Galectin-3, (B) HBME-1, (C) BRAF, and (D) Galectin-3/HBME-1 co-expression as diagnostic markers differentiating benign from malignant nodules with indeterminate FNAC samples. The red squares denote each included study, and the black diamond denotes the summarized value. The two fine dashed lines represent the 95% CI, and the red line represents the summary diagnostic performance across all included studies.

The assessment of true-positive, true-negative, false-positive, and false-negative values of Galectin-3 resulted in a pooled DOR of 20.3 (95% CI: 9.7–42.5). Derived from the pooled DOR, we calculated an AUC of 0.82 (95% CI: 0.76–0.87). Furthermore, we calculated a pooled sensitivity of 0.74 (95% CI: 0.64–0.82), a pooled specificity of 0.88 (95% CI: 0.8–0.93), a positive likelihood ratio of 5.9 (95% CI: 3.4–10.5), and a negative likelihood ratio of 0.29 (95% CI: 0.18–0.47) (Table 2, Figs 3 and 4).

**Table 2:** Meta-analysis of Galectin-3, HBME-1, BRAF, and Galectin-3/HBME-1 co-expression as diagnostic tests of indeterminate follicular nodules in FNAC.

Marker	Pooled DOR (95% CI)	Pooled AUC (95% CI)	Pooled sensitivity (95% CI)	Pooled specificity (95% CI)	Pooled pLR (95% CI)	Pooled nLR (95% CI)
Galectin-3	20.3 (9.7–42.5)	0.82 (0.76–0.87)	0.74 (0.64–0.82)	0.88 (0.8–0.93)	5.9 (3.4–10.5)	0.29 (0.18–0.47)
HBME-1	15.0 (7.3–31.0)	0.79 (0.73–0.85)	0.77 (0.67–0.84)	0.82 (0.73–0.89)	4.3 (2.5–7.3)	0.29 (0.17–0.47)
BRAF	6.6 (2.1–20.4)	0.72 (0.59–0.82)	0.17 (0.09–0.29)	0.97 (0.93–0.99)	5.7 (1.9–16.7)	0.86 (0.62–1.18)
Galectin-3 + HBME-1	45.8 (17.7–118.6)	0.87 (0.81–0.92)	0.90 (0.81–0.95)	0.84 (0.73–0.91)	5.6 (2.9–10.6)	0.12 (0.06–0.25)

DOR, diagnostic odds ratio; CI, confidence intervals; AUC, area under the curve; pLR, positive likelihood ratio; nLR, negative likelihood ratio.

The same assessment of HBME-1 resulted in a pooled DOR of 15.0 (95% CI: 7.3–31.0). Derived from the pooled DOR, we calculated an AUC of 0.79 (95% CI: 0.73–0.85). Furthermore, we calculated a pooled sensitivity of 0.77 (95% CI: 0.67–0.84), a pooled specificity of 0.82 (95% CI: 0.73–0.89), a positive likelihood ratio of 4.3 (95% CI: 2.5–7.3), and a negative likelihood ratio of 0.29 (95% CI: 0.17–0.47) (Table 2, Figs 3 and 4).

Finally, the evaluation of BRAF resulted in a pooled DOR of 6.6 (95% CI: 2.1–20.4). Derived from the pooled DOR, we calculated an AUC of 0.72 (95% CI: 0.59–0.82). Furthermore, we calculated a pooled sensitivity of 0.17 (95% CI: 0.09–0.29), a pooled specificity of 0.97 (95% CI: 0.93–0.99), a positive likelihood ratio of 5.7 (95% CI: 1.9–16.7), and a negative likelihood ratio of 0.86 (95% CI: 0.62–1.18) (Table 2, Figs 3 and 4).

Several studies (Table 1) evaluated the combined positivity of biomarkers. Only studies evaluating the combination of Galectin-3 and HBME-1 followed the criteria for inclusion. Two studies were excluded from meta-analysis since data could not be retrieved. The combined marker approach yielded a pooled DOR of 45.8 (95% CI: 17.7–118.6), a pooled AUC of 0.87 (95% CI: 0.81–0.92), a pooled sensitivity of 0.90 (95% CI: 0.81–0.95), a pooled specificity of 0.84 (95% CI: 0.73–0.91), a positive likelihood ratio of 5.6 (95% CI: 2.9–10.6), and a negative likelihood ratio of 0.12 (95% CI: 0.06–0.25) (Table 2, Figs 3 and 4).

## Discussion

In this systematic review and meta-analysis, we reviewed and summarized the existing data from studies assessing diagnostic performance of the biomarkers Galectin-3, HBME-1, and BRAF, separately, and Galectin-3/HBME-1 in combination, in evaluating the preoperative FNAC samples from patients with indeterminate cytology, classified as Bethesda category III or IV or otherwise classified indeterminate, using the postoperative histopathologic correlate as the definitive diagnosis.

FTC is a well-differentiated thyroid cancer. Currently, accurate diagnosis of benign FTAs versus malignant FTCs, which are commonly found in indeterminate categories of thyroid nodules, can be accomplished only by postoperative histopathological evaluation. FTCs are defined by capsular and/or vascular invasion, both of which are morphologic features not captured by FNAC. Due to the inability of cytology in evaluating tumor capsule integrity and vascular invasion, preoperative assessment of follicular neoplasms remains challenging, often resulting in indeterminate cytology (35).

Accurate preoperative diagnosis of either malignant or benign lesions of the thyroid gland in patients where conventional cytology is indeterminate is warranted to ensure appropriate surgical treatment and reduction in the number of diagnostic thyroidectomies (6). Therefore, the search for a biomarker or another indicator of malignancy in FNAC is warranted and ongoing. Multiple studies have evaluated Galectin-3, HBME-1, and BRAF as potential markers in improving diagnosis of malignancy in indeterminate nodules, including follicular neoplasms. No conclusion has yet been made and benign lesions with indeterminate FNAC are still being surgically removed by hemi- or total thyroidectomies, due to the lack of ability to distinguish between benign and malignant nodules preoperatively.

Galectin-3 represents one of the most studied biomarkers of indeterminate thyroid nodules, yet it has not, to our knowledge, been introduced as a standard diagnostic test for indeterminate FNAC. It plays a key role in cell growth, differentiation, apoptosis, metastasis, and malignant transformation and is known to be strongly expressed in papillary thyroid carcinoma (PTC) (36).

Among the markers analyzed in this review, Galectin-3 demonstrated the highest overall diagnostic accuracy with a DOR of 20.3 (95% CI: 9.7–42.5) and an AUC of 0.82 (95% CI: 0.76–0.87). With its high specificity of 88% (95% CI: 80–93%) and a somewhat lower sensitivity of 74% (95% CI: 64–82%), it seems more likely that benign thyroid nodules are negative for Galectin-3 expression and that positivity for Galectin-3 expression is a less sensitive marker for malignant disease. These findings align with a previous meta-analysis by Mohan *et al.* (37), reporting a pooled sensitivity of 76% and specificity of 86% for Galectin-3 expression in FNAC samples in a wider range of thyroid nodules, including Bethesda category V, ‘suspicious for malignancy’ (37).

Similarly, HBME-1, a monoclonal antibody widely used in pathology for identification of specific cell types, has proven useful in thyroid cancer diagnostics, particularly for PTC (38). In this meta-analysis, HBME-1 exhibited strong diagnostic utility, with a DOR of 15.0 (95% CI: 7.3–31.0) and an AUC of 0.79 (95% CI: 0.73–0.85), although the specificity of 82% (95% CI: 73–89%) and the sensitivity of 77% (95% CI: 67–84%) were slightly lower than that of Galectin-3. This is also consistent with previous meta-analysis by Mohan *et al.*, who found a HBME-1 specificity of 82% and sensitivity of 75% (37).

The BRAF gene encodes a protein, which regulates cell division and differentiation. Mutations in the BRAF gene have been extensively studied in thyroid cancers, especially PTC in which it has been found to be the most common genetic alteration (39). However, in this meta-analysis, BRAF mutations demonstrated a limited diagnostic performance, with the lowest DOR of 6.6 (95% CI: 2.1–20.4) and AUC of 0.72 (95% CI: 0.59–0.82). Furthermore, BRAF exhibited a very low sensitivity of 17% (95% CI: 9–29%), indicating that a considerable proportion of malignant nodules would be missed if used as a stand-alone diagnostic test. However, the high specificity of 97% (95% CI: 93–99%) supports its role as a highly predictive confirmatory marker of malignancy rather than a primary screening tool. These results are consistent with a previous meta-analysis by Su *et al.* (40), where the specificities of BRAF were 99.5 and 99.7% and sensitivities were 40.1 and 19.5% for AUS/FLUS and FN/SFN categories, respectively.

As mentioned, previous systematic reviews and meta-analyses by Mohan *et al.* (37) and Su *et al.* (40) have evaluated the diagnostic performance of Galectin-3, HBME-1, and BRAF in indeterminate nodules. However, our study differs in several important aspects. First, by employing a broader search strategy, we screened a larger amount of literature, ultimately including 23 studies for meta-analysis. Our review focused exclusively on Bethesda categories III and IV (or equivalent to these), where diagnostic uncertainty is significant due to malignancy risk estimated to be 6–40%. Prior reviews included Bethesda category V, ‘suspicious for malignancy’, with a substantially higher malignancy risk of 45–75% (6).

Furthermore, our review incorporated more recent data, thereby extending the temporal scope of previous analyses. Importantly, our study is methodologically distinct since we have utilized the SCS approach for meta-analysis. We decided to primarily use the SCS model when developing the protocol, as we expected to identify relatively few studies with the possibility of between-study heterogeneity due to both systematic errors and threshold variability. A sensitivity analysis using a conventional bivariate random-effects model has been performed and is presented in Supplementary Table S3.

Given the limitations of individual markers, several studies have investigated combined marker strategies. Five studies, evaluating co-expression of Galectin-3 and HBME-1, were suitable for meta-analysis. Dual positivity demonstrated the highest overall diagnostic performance with a pooled DOR of 45.8 (95% CI: 17.7–118.6) and an AUC of 0.87 (95% CI: 0.81–0.92), based on fewer studies than the analysis of the individual markers. Combined expression achieved a sensitivity of 90% (95% CI: 81–91%) and specificity of 84% (95% CI: 73–91%), suggesting improved diagnostic accuracy in indeterminate nodules. A study by Abram *et al.* (11), not included in the meta-analysis, similarly reported improved diagnostic performance in AUS/FLUS lesions when Galectin-3 and HBME-1 were combined.

While our meta-analysis focused on markers reported in no less than four studies, we identified additional markers, such as CK19, NRAS mutation, and PD-L1. Due to the limited number of available studies (*n* = 2–3) for certain markers, we did not include the data for these in our meta-analysis. While Cochrane guidance does not specify a specific minimum number of studies required for diagnostic meta-analysis, it emphasizes the importance of methodological robustness and careful interpretation of pooled estimates in the context of sparse data and potential heterogeneity. When only a few studies are available, Cochrane recommends considering a narrative synthesis rather than a formal meta-analysis. In line with this guidance, we opted not to include pooled estimates for these markers, as the evidence base was considered too limited to support a meaningful quantitative synthesis. A list of such markers, including their reported diagnostic performance in individual studies, is provided in Supplementary Table S1.

Moderate heterogeneity was observed for Galectin-3 (*I*^2^ = 49%) and HBME-1 (*I*^2^ = 34%). Statistical measures alone do not fully explain between-study variability in diagnostic test accuracy. Greater emphasis can be placed on methodological and clinical differences across studies. Considerable variation in staining methods, interpretation criteria such as scoring threshold, aspiration techniques, and cytology classification systems may have influenced sensitivity and specificity estimates. No statistically significant threshold effects were identified for any marker. For Galectin-3, the correlation approached statistical significance, whereas the combined Galectin-3/HBME-1 panel demonstrated a strong positive correlation (rho = 0.8). However, the estimate for the combined panel was based on only five studies and did not reach statistical significance, perhaps due to limited power. Therefore, even though it seemed that differences in positivity thresholds were unlikely to be a major source of heterogeneity for most markers, a potential threshold effect cannot be completely excluded for the combined panel. Additionally, patient population differences and study design (prospective vs retrospective) may have contributed to the variability. Furthermore, asymmetrical funnel plots suggest potential publication bias, where smaller studies with non-significant results may be underreported.

Conversely, BRAF testing exhibited no significant heterogeneity (*I*^2^ = 0%), indicating consistency among the included studies. However, asymmetry in the funnel plot suggests the presence of a small-study effect, meaning that smaller studies with fewer patients may yield more variable results. This is further supported by wide confidence intervals and a potential over- or underestimation of diagnostic performance.

Although the analysis of Galectin-3 and HBME-1, separately and combined, suggests potential diagnostic utility, diagnostic performance does not equate to proven clinical utility. The present analysis evaluates test accuracy against a histopathological reference standard but does not assess downstream clinical outcomes or reduction in unnecessary surgeries. Thus, the current evidence supports these markers as adjunctive or investigational tools rather than stand-alone decision-making tests. Further large-scale, multicenter, prospective studies are needed to determine whether improved diagnostic accuracy translates into a meaningful clinical impact. Ultimately, randomized clinical trials assessing both beneficial and harmful effects are required before implementation can be recommended.

A potential limitation is the use of postoperative histopathological diagnosis as the reference standard, thereby restricting the population to surgically treated patients. Although most patients proceeded to surgery in the studies, this design may still introduce partial verification (workup) bias, reflected in the QUADAS-2 domains of patient selection and flow and timing. As patients who underwent surgery may have a higher probability of malignancy, diagnostic accuracy estimates could be inflated, limiting generalizability to routine clinical practice.

Due to limited and inconsistently reported study-level data, subgroup analyses by cytological categories could not be reliably performed. The included studies reported indeterminate nodules inconsistently, often pooling Bethesda categories III and IV, by using other classification systems (e.g. THY3), or reporting nodules as ‘indeterminate’ without further specification. In addition, cytological classification systems evolved during the inclusion period, further limiting meaningful subgroup analyses. The present findings should be interpreted as overall estimates of biomarker performance across indeterminate thyroid nodules. The pooled diagnostic accuracy should therefore not be assumed to be directly transferable between Bethesda III and IV nodules without further category-stratified evidence. This limitation may partly reflect clinical practice in some settings, including Denmark, where management decisions for indeterminate cytology are often based on overall assessment of follicular neoplasia rather than detailed subclassification systems.

During recent years, molecular diagnostic techniques have evolved considerably*.* Molecular diagnostic platforms, including gene expression classifiers and next-generation sequencing (NGS) panels, are increasingly used in the evaluation of indeterminate thyroid nodules. These platforms typically demonstrate high sensitivity and negative predictive value and are designed to reduce unnecessary surgery (41). Contemporary molecular panels were not included in the meta-analysis since we found substantial heterogeneity in panel composition and reporting. Many studies evaluate multi-gene panels with differing combinations of mutations, gene expression profiles, or sequencing targets. As a result, pooled comparison across studies becomes methodologically challenging, particularly when raw 2 × 2 diagnostic accuracy data for individual components were not available in the studies we assessed. The rapid development of molecular diagnostic technologies may therefore limit direct comparability between studies and should be considered when interpreting the findings of this review. Future studies specifically designed to compare standardized molecular panels across comparable patient populations would be valuable to clarify their relative performance and clinical impact.

The search for preoperative, predictive factors for thyroid malignancies, in particular the FTCs, is of utmost clinical importance, which is why biomarkers and genetic findings have been evaluated intensively. Besides the markers identified in this study, several other markers, genes, and tests have been evaluated throughout the years. However, no pathognomonic biomarker or genetic finding has yet been identified. Consequently, no conclusive non-surgical test strategy for patients with indeterminate FNAC exists. Despite relatively high diagnostic values, none of the above-evaluated markers warrant immediate clinical use. To our knowledge, no studies have yet tested all three biomarkers in combination.

## Conclusion

In summary, Galectin-3 and HBME-1 appear to be the most promising biomarkers for distinguishing benign from malignant thyroid nodules in cases of indeterminate cytology, with moderate-to-high sensitivity and specificity. Their diagnostic performance may be further improved when used in combination. BRAF mutation analysis, while highly specific, lacks sufficient sensitivity to be used as a stand-alone diagnostic tool. A combination of these markers may provide the best approach to improve diagnostic accuracy and guide clinical decision-making in the management of indeterminate thyroid nodules, with the ultimate goal of reducing unnecessary diagnostic thyroidectomies. However, because category-specific analyses could not be performed, the pooled diagnostic accuracy estimates should not be assumed to be directly transferable between Bethesda III and Bethesda IV nodules without further category-stratified data.

## Supplementary materials



## Declaration of interest

The authors declare that there is no conflict of interest that could be perceived as prejudicing the impartiality of the work reported.

## Funding

This work did not receive any specific grant from any funding agency in the public, commercial, or not-for-profit sector.

## Author contribution statement

TMF conceived the study, collected the data, performed analysis, and wrote the manuscript. MHK collected the data and revised and provided final approval of the manuscript. MM performed statistical analysis and revised and provided final approval of the manuscript. PH conceived the study and revised and provided final approval of the manuscript. GBH conceived the study, collected the data, critically revised the manuscript, and supervised the project.

## Ethical approval

No ethical approval was required by this systematic review.
