# Supplementary material for: Cytological biomarkers and risk of malignancy in indeterminate thyroid nodules: a systematic review and meta-analysis
Source: Endocr Oncol. 2026 Sep 4;6(1):e250097. doi: 10.1530/EO-25-0097 (PMC13546083; doi:10.1530/EO-25-0097)
Supplement: Supplementary file 1 [file EO-25-0097_supplementary_materials.pdf]

SUPPLEMENTARY FILES

Search string Medline

Ovid MEDLINE(R) Epub Ahead of Print and In-Process, In-Data-Review & Other Non-Indexed Citations and Daily <January 16, 2025>

1. Thyroid Gland/ 61178
2. (thyroid gland* or thyroid*).ab,kf,ti. 238658
3. 1 or 2 246772
4. Biomarkers/ 376650
5. (Biochemical marker* or biologic marker* or biological marker* or biomarker* or clinical marker* or immune marker* or immunologic marker* or laboratory marker* or serum marker* or surrogate end point* or surrogate endpoint* or surrogate marker* or viral marker*).ab,kf,ti. 555565
6. 4 or 5 773989
7. Immunohistochemistry/ 305932
8. (Immunocytochemistry or immunogold silver technics or immunogold silver techniques or immunogold technic* or immunogold technique* or immunogold-silver technic or immunogold-silver technique or immunohistochemistry or immunohistocytochemistry or immunolabeling technic* or immunolabeling technique*).ab,kf,ti. 281899
9. 7 or 8 495059
10. 6 or 9 1229687
11. 3 and 10 15147

Search string Embase

Embase <1996 to 2025 Week 02> 16 jan 2025

1. Thyroid Gland/ 32581
2. (thyroid gland* or thyroid*).ab,kf,ti. 234553
3. 1 or 2 237132
4. Biomarkers/ 510693
5. (Biochemical marker* or biologic marker* or biological marker* or biomarker* or clinical marker* or immune marker* or immunologic marker* or laboratory marker* or serum marker* or surrogate end point* or surrogate endpoint* or surrogate marker* or viral marker*).ab,kf,ti. 800669
6. 4 or 5 929784
7. Immunohistochemistry/ 804851
8. (Immunocytochemistry or immunogold silver technics or immunogold silver techniques or immunogold technic* or immunogold technique* or immunogold-silver technic or immunogold-silver technique or immunohistochemistry or immunohistocytochemistry or immunolabeling technic* or immunolabeling technique*).ab,kf,ti. 404642
9. 7 or 8 885667
10. 6 or 9 1746269
11. 3 and 10 22039

Search string Cochrane

Search Name: FNA 2025
Date Run: 16/01/2025 14:34:28
Comment: Cochrane

ID	Search	Hits
1.	MeSH descriptor: [Thyroid Gland] explode all trees	721
2.	((thyroid gland* or thyroid*)):ti,ab,kw	11472
3.	#1 or #2	11472
4.	MeSH descriptor: [Biomarkers] explode all trees	32342
5.	((Biochemical marker* or biologic marker* or biological marker* or biomarker* or clinical marker* or immune marker* or immunologic marker* or laboratory marker* or serum marker* or surrogate end point* or surrogate endpoint* or surrogate marker* or viral marker*)):ti,ab,kw	99771
6.	#4 or #5	105905
7.	MeSH descriptor: [Immunohistochemistry] explode all trees	5506
8.	((Immunocytochemistry or immunogold silver technics or immunogold silver techniques or immunogold technic* or immunogold technique* or immunogold-silver technic or immunogold-silver technique or immunohistochemistry or immunohistocytochemistry or immunolabeling technic* or immunolabeling technique*)):ti,ab,kw	7198
9.	#7 or #8	10843
10.	#6 or #9	112941
11.	#3 and #10	821

**Study**	**Marker**	**Preoperative diagnosis**	**Postoperative diagnosis**	**Sensitivity (%)**	**Specificity (%)**
Abram et al. 2021 [1]	CK19	63 AUS/FLUS	45 B, 17 b	61	67
Addati et al. 2015 [2]	TROP2	12 AUS/FLUS/FN/SFN	9 B, 3 M	100	89
Barros-Filho et al. 2019 [3]	Pyro-bis	1 AUS/FLUS/FN/SFN	5 B, 5 M	80	100
Cha et al. 2018 [4]	CD56	26 AUS/FLUS	6 B, 20 M	90	83

Study	Marker	Galectin-3/HBME-1 cut-off	BRAF detection method
Abram et al. 2021	Galectin-3, HBME-1	>30% positive cells	
Addati et al. 2015	HBME-1	>70% positive cells	

Cochand-Priollet et. al 2011 [5]	HBME-1 and CK19 combined	24 AUS/FLUS/FN/SFN	13 B, 11 M	100	77
Decaussin-Petrucci et al. 2017 [6]	NRAS TERT, HRAS	61 AUS/FLUS, 124 FN/SFN	152 B, 33 M	17, 0	19, 30
Dell'Aquila et al. 2020 [7]	PD-L1	75 AUS/FLUS/FN/SFN	61 B, 14 M	71	79
Dell'Aquila et al. 2023 [8]	PD-L1	41 AUS/FLUS/FN/SFN	33 B, 8 M	75	6
Dixit et al. 2021 [9]	CD117	8 AUS/FLUS, 10 FN/SFN	7 B, 11 M	90	85
Elsharkawy et al. 2014 [10]	DNA ploidy	25 FN	13 B, 12 M	83	77
Gill et al. 2015 [11]	KRAS, NRAS, HRAS	60 AUS/FLUS/FN/SFN	37 B, 23 M	9, 9, 17,4	84, 100, 95
Gotnayer et al. 2023 [12]	Zinc	16 AUS/FLUS/FN/SFN	5 B, 11 M	100	80
Lastra et al. 2014 [13]	Afirma mRNA	50 AUS/FLUS/FN/SFN	28 B, 22 M	100	7
Leslie et al. 2015 [14]	CK19	8 AUS/FLU/SFN/SFN	1 B, 7 M	29	100
Mazeh et al. 2013 [15]	miRNA	6 AUS/FLUS/FN/SFN	2 B, 4 M	100	75
Policardo et al. 2023 [16]	P53	48	43 B, 5 M	60	95
Rodriguez-Rodero et al. 2022 [17]	Three CPG positions	9 AUS/FLUS/FN/SFN	4 B, 5 M	80	100
Rossi et al. 2005 [18]	RET	37 follicular lesions*	22B, 15 M	80	55
Rossi et al. 2018 [19]	MiR-375	37 AUS/FLUS and FN/SFN	30 B, 7 M	88	65
Torregrossa et al. 2010 [20]	CXCR4	43 indeterminate*	28 B, 15 M	80	93
Xu et al. 2022 [21]	QCIGISH - SNRPN OG HM13	81 AUS/FLUS/FN/SFN	25 B, 56 M	0	100
Yang et al. 2016	GEC	67 AUS/FLUS/FN/SFN	35 B, 32 M	0	100

Table S1 Data not included in meta-analysis since markers appear in less than four studies. Molecular biomarkers, preoperative fine needle aspiration cytology and final histopathological benign or malignant diagnosis of indeterminate thyroid nodules.

*Not further specified.

AUS: atypia of undetermined significance, B: benign, FLUS: follicular lesion of undetermined significance, FN: follicular neoplasm, M: malignant, SFN: suspicious of follicular neoplasm

Anand et al. 2021	BRAF		Immunocytochemistry*
Barros-Filho et al. 2019	BRAF		PCR followed by pyrosequencing
Bartolazzi et al. 2008	Galectin-3	>5% positive cells	
Borrelli et al. 2016	BRAF		High resolution melt + Sanger sequencing (Easy BRAF real-time PCR)
Cha et al. 2018	HBME-1	>30% positive cells	
Collet et al. 2005	Galectin-3	>50% positive cells	
Decaussin-Petrucci et al. 2017	BRAF		High-resolution melting + Sanger sequencing
Dell'Aquila et al. 2020	BRAF		Not specified
Dixit et al. 2021	Galectin-3	Immunocytochemistry score ≥2 **	
Elsharkawy et al. 2014	Galectin-3	>10% positive cells	
Fadda et al. 2011	Galectin-3, HBME-1	>50% positive cells	
Gill et al. 2015	BRAF		PCR followed by pyrosequencing
Leslie et al. 2015	HBME-1, BRAF	Positive when accentuated membranous staining was present	Allele-specific real-time PCR); SSCP for equivocal/discordant cases
Mills et al. 2005	Galectin-3	Not specified	
Pennelli et al. 2009	Galectin-3	>10% positive cells	
Rossi et al. 2005	Galectin-3, HBME-1	>50% positive cells	
Rossi et al. 2016	Galectin-3. HBME-1	>50% positive cells	
Rossi et al. 2018	Galectin-3, HBME-1, BRAF	>50% positive cells	PCR followed by pyrosequencing
Sapio et al. 2007	Galectin-3, BRAF	>10% positive cells	Mutant allele-specific PCR
Torregrossa et al. 2010	Galectin-3, HBME-1	>10% positive cells	
Zhang et al. 2015	Galectin-3, HBME-1	Not specified	

Table S2 Immunocytochemistry positivity criteria for Galectin-3 and HBME-1 and BRAF mutation detection methods used in the studies included in the meta-analysis.

* Cytoplasmic staining intensity 2+ or 3+ considered positive; 0 or 1+ considered negative. the absence of staining was scored 0, weak cytoplasmic staining was scored 1+, moderate cytoplasmic staining was scored 2+, and intense cytoplasmic staining was scored 3+.

** Calculated as percentage score (<5%=0, 5–50%=1, >50%=2) plus intensity score (weak=1, moderate=2, strong=3)

PCR: polymerase chain reaction, SSCP: Single-strand conformation polymorphism

Marker	Pooled Sensitivity (95% CI)	Pooled Specificity (95% CI)	Pooled AUC
Galectin-3	0.69 (0.55-0.81)	0.84 (0.77-0.90)	0.85
HBME-1	0.75 (0.61-0.86)	0.80 (0.73-0.86)	0.85
BRAF	0.15 (0.09-0.25)	0.97 (0.93-0.99)	0.71
Galectin-3+HBME-1	0.84 (0.70-0.93)	0.83 (0.7-0.91)	0.90

Table S3 Sensitivity-analysis of Galectin-3, HBME-1 and BRAF as diagnostic tests of indeterminate follicular nodules in FNAC using a conventional bivariate model.

CI: confidence intervals, AUC: area under the curve

References

1. Abram M, Huhtamella R, Kalfert D, Hakso-Makinen H, Ludvikova M, Kholova I. The Role of Cell Blocks and Immunohistochemistry in Thyroid Atypia of Undetermined Significance/Follicular Lesion of Undetermined Significance Bethesda Category. *Acta cytologica*. 2021;65(3):257-263. doi:10.1159/000514906

2. Addati T, Achille G, Centrone M, et al. TROP-2 expression in papillary thyroid cancer: a preliminary cyto-histological study. *Cytopathology : official journal of the British Society for Clinical Cytology*. 2015;26(5):303-311. doi:10.1111/cyt.12196

3. Barros-Filho MC, Dos Reis MB, Beltrami CM, et al. DNA Methylation-Based Method to Differentiate Malignant from Benign Thyroid Lesions. *Thyroid : official journal of the American Thyroid Association*. 2019;29(9):1244-1254. doi:10.1089/thy.2018.0458

4. Cha H, Pyo JY, Hong SW. The Usefulness of Immunocytochemistry of CD56 in Determining Malignancy from Indeterminate Thyroid Fine-Needle Aspiration Cytology. *Journal of pathology and translational medicine*. 2018;52(6):404-410. doi:10.4132/jptm.2018.09.20

5. Cochand-Priollet B, Dahan H, Laloi-Michelin M, et al. Immunocytochemistry with cytokeratin 19 and anti-human mesothelial cell antibody (HBME1) increases the diagnostic accuracy of thyroid fine-needle aspirations: preliminary report of 150 liquid-based fine-needle aspirations with histological control. *Thyroid : official journal of the American Thyroid Association*. 2011;21(10):1067-1073. doi:10.1089/thy.2011.0014

6. Decaussin-Petrucci M, Descotes F, Depaepe L, et al. Molecular testing of BRAF, RAS and TERT on thyroid FNAs with indeterminate cytology improves diagnostic accuracy. *Cytopathology : official journal of the British Society for Clinical Cytology*. 2017;28(6):482-487. doi:10.1111/cyt.12493

7. Dell'Aquila M, Granitto A, Martini M, et al. PD-L1 and thyroid cytology: A possible diagnostic and prognostic marker. *Cancer cytopathology*. 2020;128(3):177-189. doi:10.1002/cncy.22224

8. Dell'Aquila M., Tralongo P., Granitto A., et al. Update regarding the role of PD-L1 in oncocytic thyroid lesions on cytological samples. *J Clin Pathol*. 2023;76(10):671 EP - 677. doi:10.1136/jclinpath-2022-208215

9. Dixit S, Diwaker P, Wadhwa N, Arora VK. Galectin-3 and CD117 immunocytochemistry in the diagnosis of indeterminate thyroid lesions: A pilot study. *Diagnostic cytopathology*. 2021;49(10):1129-1137. doi:10.1002/dc.24835

10. Elsharkawy SL, AbdEl-Aal WE, Esmal RSE, Ali HHM, Mahfouz SM, El-Habashi A. Preoperative evaluation of thyroid epithelial lesions by DNA ploidy and galectin-3 expression in FNAC. *Open Access Macedonian Journal of Medical Sciences*. 2014;2(4):585-594. doi:10.3889/oamjms.2014.105

11. Gill MS, Nayan S, Kocovski L, et al. Local molecular analysis of indeterminate thyroid nodules. *Journal of otolaryngology - head & neck surgery = Le Journal d'oto-rhino-laryngologie et de chirurgie cervico-faciale*. 2015;44:52-52. doi:10.1186/s40463-015-0106-2

12. Gotnayer L., Aranovich D., Fraenkel M., Yoel U., Vidavsky N. Zinc in microscopic calcifications isolated from thyroid fine needle aspiration may serve as a biomarker of thyroid nodule malignancy: A promising proof-of-concept. *Acta Biomater*. 2023;161((Gotnayer, Aranovich, Vidavsky) Department of Chemical Engineering, Ben-Gurion University of the Negev, Beer Sheva, Israel):275 EP - 284. doi:10.1016/j.actbio.2023.03.010

13. Lastra RR, Pramick MR, Crammer CJ, LiVolsi VA, Baloch ZW. Implications of a suspicious afirma test result in thyroid fine-needle aspiration cytology: an institutional experience. *Cancer cytopathology*. 2014;122(10):737-744. doi:10.1002/cncy.21455

14. Leslie C, Grieu-Iacopetta F, Richter A, et al. BRAF p.Val600Glu (V600E) mutation detection in thyroid fine needle aspiration cell block samples: a feasibility study. *Pathology*. 2015;47(5):432-438. doi:10.1097/PAT.0000000000000273

15. Mazeh H, Levy Y, Mizrahi I, et al. Differentiating benign from malignant thyroid nodules using micro ribonucleic acid amplification in residual cells obtained by fine needle aspiration biopsy. *Journal of Surgical Research*. 2013;180(2):216-221. doi:10.1016/j.jss.2012.04.051

16. Policardo F., Tralongo P., Arciuolo D., et al. p53 expression in cytology samples may represent a marker of early-stage cancer. *Cancer Cytho*. 2023;131(6):392 EP - 401. doi:10.1002/cncy.22694

17. Rodriguez-Rodero S, Morales-Sanchez P, Tejedor JR, et al. Classification of follicular-patterned thyroid lesions using a minimal set of epigenetic biomarkers. *European journal of endocrinology*. 2022;187(3):335-347. doi:10.1530/EJE-22-0012

18. Rossi ED, Raffaelli M, Minimo C, et al. Immunocytochemical evaluation of thyroid neoplasms on thin-layer smears from fine-needle aspiration biopsies. *Cancer*. 2005;105(2):87-95.

19. Rossi ED, Martini M, Capodimonti S, et al. Morphology combined with ancillary techniques: An algorithm approach for thyroid nodules. *Cytopathology : official journal of the British Society for Clinical Cytology*. 2018;29(5):418-427. doi:10.1111/cyt.12555

20. Torregrossa L, Faviana P, Filice ME, et al. CXC chemokine receptor 4 immunodetection in the follicular variant of papillary thyroid carcinoma: Comparison to galectin-3 and hector battifora mesothelial cell-1. *Thyroid*. 2010;20(5):495-504. doi:10.1089/thy.2009.0282

21. Xu H, Zhang Y, Wu H, et al. High Diagnostic Accuracy of Epigenetic Imprinting Biomarkers in Thyroid Nodules. *Journal of clinical oncology : official journal of the American Society of Clinical Oncology*. 2023;41(6):1296-1306. doi:10.1200/JCO.22.00232
